# Digital Storytelling to Communicate COVID-19 Research Efforts

**DOI:** 10.46804/2641-2225.1208

**Published:** 2025-03-08

**Authors:** Carolyn E. Gray, Kelly Waters, Kathyrn Bizier, Brenda M. Joly

**Affiliations:** a Catherine Cutler Institute, Muskie School of Public Service, University of Southern Maine, Portland, Maine; b Public Health Program, Muskie School of Public Service, University of Southern Maine, Portland, Maine

**Keywords:** COVID-19, Research, Information dissemination, Program evaluation

## Abstract

**Problem statement:**

COVID-19 was a once-in-a-century pandemic that hit the world in March 2020. Overnight, the SARS-CoV-2 virus threatened not only survival, but also food security, social supports, transportation issues, financial constraints, and more. Timely research became crucial to understanding how to mitigate viral spread, treat patients, and address the consequences and health implications.

**Background:**

Advancing the infrastructure to support research in northern New England has been the focus of the Northern New England Clinical and Translational Research (NNE-CTR) Network since 2017. The NNE-CTR Network is a multi-year initiative in Maine, Vermont, and New Hampshire that brings together academic institutions, health care organizations, and local community stakeholders to foster new research and address health challenges unique to the northeast region. The NNE-CTR Network was well-positioned to help address COVID-19, not only locally, but also nationally and worldwide.

**Application:**

The NNE-CTR Network’s Tracking and Evaluation Core (TEC) assessed the NNE-CTR Network’s COVID-19 research during the first 3 years of the pandemic. Using an innovative, arts-based dissemination method known as digital storytelling, the TEC summarized and shared the NNE-CTR Network’s immediate and broad response to the pandemic. The analysis revealed 4 major ways the NNE-CTR Network responded to the pandemic: (1) quickly reallocating funding for early pandemic research, (2) providing innovative laboratory support and technologies, (3) collaborating in national research efforts, and (4) conducting research on a range of pandemic topics. The TEC used digital storytelling to highlight 18 research projects that addressed the pandemic.

## 1. Problem statement

COVID-19 was a once-in-a-century pandemic that hit the world in March 2020. Overnight, the SARS-CoV-2 virus became a threat to humanity, disrupting nearly all aspects of life, including health care delivery. The need for timely and responsive research during the pandemic was crucial.^1^ However, historically, it has been challenging to quickly pivot, implement new research efforts, and share those efforts in a timely manner to promote adoption and translation to practice. ^2^

## 2. Background

The Northern New England Clinical and Translational Research (NNE-CTR) Network began in 2017 with funding from the National Institutes of Health. This multi-year initiative in Maine, Vermont, and New Hampshire brings together a network of academic institutions, health care organizations, and local community stakeholders to foster new research and address health challenges, including those unique to the northeast region.^3^ As of June 2024, a total of 795 individuals registered with the NNE-CTR Network. Registrants are largely clinicians and researchers, including early-career scientists looking to strengthen their research skills.^4^ The NNE-CTR Network supports investigators through professional development, mentorship, research navigation, translational technology services and supports, as well as pilot research funding. As of September 2023, the NNE-CTR Network has funded 40 pilot research projects that cover a range of issues. Given the aims of the NNE-CTR Network, this network was in a strong position, with experienced leadership in the administrative core, to swiftly adapt their work to help address COVID-19, not only locally, but also nationally and worldwide.

## 3. Application

The NNE-CTR Network’s Tracking and Evaluation Core (TEC) includes a group of researchers and evaluation specialists whose primary responsibility is to evaluate the initiative.^5^ The TEC assessed the NNE-CTR Network’s response to the pandemic from 2020 to 2023 using a mixed-methods approach (Table 1). Quantitative and qualitative data were gathered by systematically identifying and cataloging all COVID-19–related efforts into an inventory based on existing administrative data sources (eg, pilot award applications). Document reviews were based on COVID-19–related project reports, funding applications, publications, media events, and other products related to the research. Results from this inventory were summarized using digital storytelling to disseminate findings. Digital storytelling is an innovative evaluation tool that can be used to share evaluation findings.^6^

To compile this inventory, the TEC team reviewed all COVID-19–related pilot research projects, publications, media, and presentations supported by the NNE-CTR Network during this timeframe. The inventory recorded: (1) the category of research; (2) the topic and focus; (3) the research team, target population, and timing or status; and (4) articles, presentations, and any press or news events. Interviews were conducted with the NNE-CTR Network’s leadership about other COVID-19-specific efforts supported by the NNE-CTR Network. Decisions about which stories to include in the digital story involved inclusion criteria of having a clear NNE-CTR Network connection, a clear COVID-19 focus, and enough data available, as well as being interesting to a wide audience. Exclusion criteria included having a limited NNE-CTR Network connection and not having enough data available. The TEC, in consultation with NNE-CTR Network leadership, selected 18 of 26 research projects to highlight via digital storytelling, and 3 researchers agreed to participate in key informant interviews to highlight their innovative efforts (Table 2). Six of these projects were pilot research projects funded by the NNE-CTR Network. Quotes and video clips from the interview recordings were identified by the TEC team for inclusion in the digital story.

### 3.1. Assessment results

The NNE-CTR Network’s response to COVID-19 was immediate and broad. The NNE-CTR Network responded to the pandemic by quickly reallocating funding to support early pandemic research projects; supporting innovative technologies by using the NNE-CTR Network’s existing infrastructure; contributing to national research efforts, such as the development of a national clinical data resource that can be used to study COVID-19; and conducting innovative research to rapidly tackle urgent COVID-19 issues. The NNE-CTR Network’s response included more than 20 different COVID-19 research topics, from vaccine hesitancy and ventilators to telemedicine and testing.

Using StoryMaps^7^ as a platform to show digital stories, 6 themes were identified to provide research spotlights about the 18 selected projects that show the NNE-CTR Network’s response to the pandemic. These themes included: (1) new technologies, (2) funded projects, (3) national efforts, (4) community projects, (5) rural testing, and (6) food security. This collection of digital stories can be viewed at https://arcg.is/0SCSm5.

#### 3.1.1. New technologies

The research highlighted in this digital story focused on the innovative technologies supported by the NNE-CTR Network to address the need for ventilators, viral variant sequencing, and mask sterilization. The number of patients with COVID-19 coupled with the limited supply of ventilators led to the development and testing of a simple, inexpensive, emergency ventilator for patients with COVID-19, called “The Vermontilator.” The Vermontilator was just as effective as a regular ventilator and was developed using a laboratory supported by the NNE-CTR Network’s Translational Research Technologies Core. As new viral variants emerged and the need for testing grew, the NNE-CTR Network’s Translational Research Technologies Core supported the development of rapid, single-step preparations of patient specimens for sequence-based determination of the virus and viral variants. In addition, the need for an automated system to sterilize N95 masks was apparent early during the COVID-19 pandemic as cases rose. An NNE-CTR Network investigator developed a portable system to inactivate viral particles on N95s, termed the UV LAVE.

#### 3.1.2. Funded projects

The NNE-CTR Network provided funding for 6 pilot projects that focused on a variety of pandemic-related issues. These studies largely involved multiple institutions and engaged a total of 41 researchers. The projects focused on how to communicate health information, the impacts of social distancing for vulnerable populations (eg., older adults, people who inject drugs), the impact of hospital visitation restrictions on caregivers and patients, variation in immune response to SARS-CoV-2, and vaccine hesitancy. This work was disseminated through publications, presentations, and NNE-CTR Network communications.

#### 3.1.3. National efforts

In keeping with the mission of clinical and translational research efforts funded by the National Institutes of Health, efforts to develop and share research tools and data were promptly initiated. The NNE-CTR Network played an active and lead role in the National COVID Cohort Collaborative (N3C).^8^ The N3C Data Enclave stores one of the largest collections of data on patients with COVID-19 and is a product of more than 75 institutions, making it the one of the largest publicly available electronic health records limited data sets in US history. This data enclave provides a centralized location for data access that can be used in research on COVID-19 and identifying potential treatments. The University of Vermont and MaineHealth (an integrated health system with Maine’s largest medical group in northern New England)^9^ are major contributors and users of the N3C Data Enclave. This centralized collection of data has helped drive leading research on the epidemiology and pathophysiology of COVID-19.

The NNE-CTR Network also played a key role in other national projects: Researching COVID to Enhance Recovery (RECOVER),^10^ Pathobiology in RECOVER of Metabolic and Immune Systems (PROMIS), and Rapid Acceleration of Diagnostics-Underserved Populations (RADx-UP).^11,12^ The RECOVER initiative is a multi-year longitudinal study of patients with long COVID. Work continues within the NNE-CTR Network to identify the underlying cause and potential treatments for this condition. For example, the PROMIS study (part of the national RECOVER initiative) is investigating the SARS-CoV-2 virus in people with long COVID. The RADx-UP research projects were part of a national effort designed to study a variety of COVID-19 testing methods and approaches. The NNE-CTR Network was selected to help lead 2 local studies in northern New England. One project focused on testing in an urban setting (highlighted in the community projects research spotlight), and the other focused on rural communities (highlighted in the rural testing research spotlight).

#### 3.1.4. Community projects

Locally, the NNE-CTR Network supported several projects in Maine, New Hampshire, and Vermont to learn about vaccine hesitancy, food security for prenatal women, and stress among front-line workers. Another project funded by RADx-UP focused on providing COVID-19 testing to immigrant, low-income, and unhoused populations around Portland, Maine. Another example is the Northern New England CO-OP Practice and Community Based Research Network (NNE CO-OP PCBRN), a partner of the NNE-CTR Network, that studies the factors influencing screening for food security during prenatal appointments.

#### 3.1.5. Rural testing

Given the rurality in Maine and Vermont, NNE-CTR Network researchers were well-positioned to explore COVID-19 testing in rural areas. Researchers received RADx-UP funding to study the factors influencing COVID-19 testing, including the barriers and communication strategies perceived as most effective. Engagement with community partners was a key element of this research.

#### 3.1.6. Food security

When the pandemic hit, the issue of food security became an even more complex issue. In the first month of the pandemic, an NNE-CTR Network researcher started studying the impact of COVID-19 on food access, food security, and food systems. The work led to the development of a survey instrument that was widely shared with other researchers and helped to standardize data-collection efforts. By July 2020, the research led to the engagement of 18 study sites and the formation of the National Food Access and COVID Research Team. This consortium now has 20 study sites and continues to evaluate the ongoing problem of food security due to the pandemic and how health outcomes have been impacted.

### 3.2. Digital storytelling

The application of digital storytelling to showcase the NNE-CTR Network’s COVID-19 response was well regarded by NNE-CTR Network leadership, and, recently, evaluators have been called on to consider adopting this approach.^6^ The StoryMaps platform allowed evaluators to summarize and disseminate the NNE-CTR Network’s research using this innovative approach and a storytelling framework. By including first-hand accounts from researchers, pictures of their efforts, and videos and quotes reflecting on their work, the TEC was able to share the NNE-CTR Network’s response with a broad audience using a non-traditional approach. As funders and the public increasingly call for evidence and impact stories, this novel approach may garner more attention.

## 4. Conclusion

Our assessment of the NNE-CTR Network’s response to COVID-19 revealed a network that quickly pivoted to address pressing health issues through research that was timely, relevant to northern New England, and often contributed to efforts beyond the original scope. Our digital storytelling approach provided a creative method for sharing the evaluation findings beyond typical publications and reports. Through evidence of publications, presentations, and collaboration efforts, the NNE-CTR Network’s research likely contributed to understanding and addressing COVID-19–related topics locally, nationally, and worldwide. The digital stories provided a creative method for highlighting COVID-19–related research that may extend beyond the academic, clinical, and research environment.

## Figures and Tables

**Table 1.** Digital storytelling process for NNE-CTR COVID-19 research.

Step	Description
1. Systematically identified and cataloged all COVID-19–related efforts into an inventory	Reviewed data sources, including pilot research projects, publications citing the NNE-CTR Network, media, and presentations supported by the NNE-CTR Network from March 2020 through December 2023 Inventory recorded category of research, topic/focus, population, and data sources Initially had 26 research projects
2. Selected final research projects to highlight via digital storytelling	Tracking and Evaluation Core selected 18 research projects in consultation with NNE-CTR Network leadership Inclusion criteria: clear NNE-CTR Network connection, clear COVID-19 focus, enough data available, and interesting to a wide audience Exclusion criteria: limited NNE-CTR Network connection and not enough data available
3. Summarized findings/content	Identified 4 key themes Created storyboard to plan how to share themes and individual project stories within larger digital story collection
4. Gathered additional materials to use in the digital story	Recorded new video of project leads for RADx-UP vaccination hesitation project, Vermont food-security survey, and NNE-CTR Network leadership (for background/context) in collaboration with the MaineHealth marketing team and University of Vermont studio Selected clips from existing video recordings (eg, NNE-CTR Network seminars) and audio recordings (eg, media interviews) Requested photos and additional context from researchers Drafted/revised initial digital stories
5. Finalized and disseminated digital story collection	Soft launch with all researchers included in digital stories NNE-CTR Network leadership approved final collection Disseminated collection via the NNE-CTR Network’s website, newsletter, and email listserv Researchers involved were encouraged to share the link

Abbreviations: NNE-CTR, Northern New England Clinical and Translational Research; RADx-UP, Rapid Acceleration of Diagnostics-Underserved Populations.

**Table 2.** Research projects included in final digital story collection, as of December 2023.

Project focus area	NNE-CTR connection	Population/Scope	Result highlights	Data sources sources available
New technologies				
Vermontilator	Supported by Translational Research Technologies Core	Population: NA Scope: Global	Developed prototype of emergency ventilator in <1 month Total of 15 parts to make (vs 1500 parts for a typical ventilator) Costs <10% less than a typical ventilator (~$2000 vs ~$25000)	Presentations Media
Variant sequencing	Equipment purchased by the NNE-CTR Network	Population: NA Scope: Global	Developed rapid, single-step preparations of patient specimens for sequence-based determination of SARS-CoV-2 (faster testing results) <2 months to have a laboratory up and running	Publications Presentations
Mask sterilization	NNE-CTR Network member	Population: NA Scope: Global	Developed portable system to inactivate viral particles on N95s Cost-effective and environmentally friendly alternative to buying more N95s	Presentations Media Other
Funded projects				
Health communication	Direct funding from the NNE-CTR Network	Population: National sample of 1500 US adults Scope: National	“Uncertainty-normalizing” communication strategies reduced some aversive psychosocial responses Strategies promoting hope and prosocial values did not have that same effect	Publications Other
Social isolation (older adults)	Direct funding from the NNE-CTR Network	Population: 225 older adults in Maine (survey), 30 follow-up interviews Scope: State (Maine)	Helpful coping themes emerged: remembering the need to isolate is temporary, staying busy, regulating information intake Other findings: older adults were sleeping more poorly and exercising less during the pandemic	Other
Social isolation (people who inject drugs)	Direct funding from the NNE-CTR Network	Population: 36 key stakeholders in Maine Scope: State (Maine)	Relaxing needle exchange policies helped people who inject drugs access needed harm-reduction services	Publications Presentations Media Other
Remote caregivers	Direct funding from the NNE-CTR Network	Population: 200 patients across MaineHealth (medical records), 13 interviews Scope: State (Maine)	Visitor restrictions significantly reduced the amount of communication between caregivers and patients’ medical teams, which led to emotional distress in both caregivers and patients	Publications Other
Immune response to COVID-19	Direct funding from the NNE-CTR Network	Population: 26 patients with COVID-19 Scope: State (Maine)	A certain subset of white blood cells might be important to target to reduce secondary tissue damage	Publications Media Other
Vaccine hesitancy	Direct funding from the NNE-CTR Network	Population: 148 Maine residents Scope: State (Maine)	Respondents who were vaccine-hesitant questioned efficacy of vaccines and worried about side effects Younger respondents were more likely to be vaccine-hesitant No differences between residents living in metro vs rural areas Health care providers were viewed as trusted sources for information about vaccines	Publications Presentations Media Other
National efforts				
National COVID Cohort Collaborative (N3C)	NNE-CTR Network collaborated with other CTRs*/CTSAs^†^ to develop the N3C platform; ongoing involvement	Population: Electronic health records from >22 million patients, including almost 9 million with COVID-19 Scope: National	Largest electronic health records data set in US history Centralized collection of data has helped drive leading research on the epidemiology and pathophysiology of COVID-19	Publications Presentations Media Other
RECOVER (Long COVID)	Funded in collaboration with other CTRs*	Population: Data from 9764 participants (including 119 from MaineHealth) Scope: National	With post–COVID-19 condition, certain symptoms tend to manifest together There were differences before and after the 2021 Omicron variant (post–COVID-19 condition was more common and more severe)	Publications Presentations Media Other
PROMIS (Long COVID)	Funded in collaboration with other CTRs*	Population: NA (ongoing) Scope: National	NA (ongoing)	Media Other
Community projects				
RADx-UP (Portland)	Principal Investigator is a co-lead in the NNE-CTR Network Community Engagement and Outreach Core	Population: 217 people who attended walk-up testing clinics in Portland, Maine Scope: Community	Low-barrier testing is important for reducing the spread of SARS-CoV-2 and reaching vulnerable populations Potential to reduce disparities in access to antiviral treatments	Publications Presentations Media Other
Food security screening	NNE CO-OP PCBRN research	Population: 42 primary care/prenatal care practices in Maine, New Hampshire, and Vermont Scope: Regional (northern New England)	Respondents who reported new food support programs in their communities were more likely to have greater confidence in their primary care practice and community capacity to address food insecurity	Publications Other
Rural vaccine hesitancy	NNE CO-OP PCBRN research	Population: Patients at 3 primary care practices in Maine, New Hampshire, and Vermont Scope: Regional (northern New England)	NA (ongoing)	Presentations Other
Health care worker resiliency	NNE CO-OP PCBRN research	Population: 40 health care sites in Maine, New Hampshire, and Vermont Scope: Regional (northern New England)	NA (ongoing)	Publications Other
Rural testing				
RADx-UP – COVID-19 testing in rural populations	Principal investigator is co-lead in NNE-CTR Network Community Engagement and Outreach Core	Population: 90 participants in rural Vermont and Maine Scope: Regional (northern New England)	Respondents reported testing because they wanted to keep others safe, were worried about severe illness, and were required to test for work or school Testing barriers included accessing test sites, changing rules and communications about testing over time, and the financial costs related to positive tests (time away from work)	Presentations Media Other
Food security				
Impact of COVID-19 on food security	Direct funding from the NNE-CTR Network and Gund Institute	Population: 3200 Vermont residents Scope: State (Vermont)	Respondents reported a 33% increase in food insecurity across the state in initial survey Original survey was shared with other researchers	Publications Presentations Other

Abbreviations: NA, not applicable; NNE CO-OP PCBRN, Northern New England CO-OP Practice and Community Based Research Network; NNE-CTR, Northern New England Clinical and Translational Research Network; PROMIS, Pathobiology in RECOVER of Metabolic and Immune Systems; RADx-UP; Rapid Acceleration of Diagnostics-Underserved Populations; RECOVER, Researching COVID to Enhance Recovery.

* Institutional Development Award Networks for Clinical and Translational Research (IDeA-CTR) grants.

† Clinical and Translational Science Awards (CTSA) Program.
